# Supplementary material for: Pathogenicity of PKCγ Genetic Variants—Possible Function as a Non-Invasive Diagnostic Biomarker in Ovarian Cancer
Source: Genes (Basel). 2023 Jan 16;14(1):236. doi: 10.3390/genes14010236 (PMC9858858; doi:10.3390/genes14010236)
Supplement: Supplementary file 1 [file genes-14-00236-s001.zip › genes-2143753-Supplementary material.pdf]

Supplementary Material

Pathogenicity of PKCγ Genetic Variants - possible function as a non-invasive diagnostic biomarker in Ovarian Cancer

Kanza Shahid[1], Khushbukhat Khan[1], Yasmin Badshah[1], Naeem Mahmood Ashraf[2], Arslan Hamid[3], Janeen H. Trembley[4,5,6], Maria Shabbir[1*], Tayyaba Afsar[7], Ali Almajwal[7], Ali Abusharha[8,] Suhail Razak[4*]

Table S1: Genotypic distribution of A24S (rs923331350) SNP in Ovarian Cancer

**Genotype**	**Patient (n= 49)**	**Control (n=51)**	**Odds Ratio**	**95% CI – odds ratio**	**Relative Risk**	**95% CI – relative risk**	**P Value**
	N, (%)	N, (%)					
**GG**	0<br>0%	0<br>0%	___	___	___	___	>0.9999
**AG**	49<br>100%	51<br>100%	___	___	___	___	>0.9999
**AA**	0<br>0%	0<br>0%	___	___	___	___	>0.9999

Table S2: Docking Parameters of Wild and Mutant Proteins

Docking Parameters	PKCG_Wild	A24S Mutant	K359R Mutant
**HADDOCK score**	-9.3 +/- 5.7	-2.1 +/- 14.8	-23.6 +/- 3.4
**Cluster size**	28	8	7
**RMSD from the overall lowest-energy structure**	19.0 +/- 0.3	20.3 +/- 0.3	22.2 +/- 0.1
**Van der Waals energy**	-83.6 +/- 8.5	-63.0 +/- 9.1	-122.0 +/- 13.6
**Electrostatic energy**	-198.0 +/- 36.7	-471.6 +/- 67.9	-238.8 +/- 52.6
**Desolvation energy**	-6.5 +/- 2.7	-3.4 +/- 8.2	-16.9 +/- 5.8
**Restraints violation energy**	1205.0 +/- 105.30	1585.6 +/- 182.5	1631.2 +/- 161.16
**Buried Surface Area**	2742.2 +/- 147.0	2992.5 +/- 341.0	3631.2 +/- 162.4
**Z-Score**	-2.0	-1.8	-2.2

Table S3: Data of each participant from diseased group regarding the clinical features along with [illegible]

Sample ID	Clinicopathological features Stage I	II	III	IV	Metastatic	Genotype GG	AA	GA
ASAB-KS-01				1	1	1		
ASAB-KS-02				1	0			1
ASAB-KS-03		1			0	1		
ASAB-KS-04			1		0			1
ASAB-KS-05			1		1		1	
ASAB-KS-06			1		0			1
ASAB-KS-07	1			1	1	1		
ASAB-KS-08				1	1	1		
ASAB-KS-09			1		0			1
ASAB-KS-10					0	1		
ASAB-KS-11			1		0			1
ASAB-KS-12		1			1		1	
ASAB-KS-13		1			1		1	
ASAB-KS-14			1		1		1	
ASAB-KS-15		1			1		1	
ASAB-KS-16	1				0			1
ASAB-KS-17	1				1		1	
ASAB-KS-18				1	0	1		
ASAB-KS-19		1			0			1
ASAB-KS-20			1		1		1	
ASAB-KS-21	1				0	1		
ASAB-KS-22		1			1		1	
ASAB-KS-23			1		0			1
ASAB-KS-24		1			1			1
ASAB-KS-25			1		1		1	
ASAB-KS-26			1		0	1		
ASAB-KS-27				1	0	1		
ASAB-KS-28				1	0	1		
ASAB-KS-29		1			0		1	
ASAB-KS-30		1			1		1	
ASAB-KS-31				1	0	1		
ASAB-KS-32			1		1			1
ASAB-KS-33				1	1		1	
ASAB-KS-34				1	0		1	
ASAB-KS-35				1	0		1	
ASAB-KS-36			1		0			1
ASAB-KS-37	1				0		1	
ASAB-KS-38				1	1			1
ASAB-KS-39				1	0		1	
ASAB-KS-40				1	0	1		
ASAB-KS-41			1		1			1
ASAB-KS-42				1	0		1	

ASAB-KS-43	1		1		1	
ASAB-KS-44		1	0			1
ASAB-KS-45	1		0		1	
ASAB-KS-46		1	0			1
ASAB-KS-47	1		0		1	
ASAB-KS-48		1	0	1		
ASAB-KS-49		1	0	1		

Table S4: Patient Consent Form

Cancer Associated Pathogenic Variant of KPC family

Project Title: Possible Prognostic Impact of KPCI, KPCE, KPCG Genetic Variant in Prostate, Cervical and Ovarian cancer

IRB Number: __________

Participant ID: ________________

Participants Name: _____________

Principle Investigator: Dr Maria Shabbir

Study Purpose:

Study aims to identify genetic variation in KPC family genes that are associated with cancer in Pakistani Population. Identification of polymorphism will help in pre-diagnosis and prognosis of the disease. Information collected in the study will be kept confidential and blood sample collected in the study will solemnly be used for research purpose.

PI: ___________________

For Donors:

I voluntarily agree to take part in this research project. I have been informed how and where my sample will be used. My blood sample may be collected and used in this study as defined in this consent form. Aim of this study has been conveyed to me in my native language too.

Donor's Signature: _________________ *Date:* __________________

ASAB

ATTA-UR-RAHMAN SCHOOL OF APPLIED BIOSCIENCES
NATIONAL UNIVERSITY OF SCIENCES & TECHNOLOGY

Table S5: Patient History Form

Patient details

Name ________________________________ Age ______________________________

Gender: Male / Female Marital status: Single/Married

Ethnic background ___________________ Education: __________________________

Please select from chart below:

Cancer localization				
Cancer type	Primary		Secondary	
Cancer metastasis	Yes		No	
Cancer stage	I	II	III	IV
Treatment status	Treated		Not treated	
If yes, duration of treatment				
Family History	Yes		No	
If Yes, please state the name of cancer: _____________________________				
Relationship with the patient:________________________________				
Smoking	Yes		No	
Alcohol/drug consumption	Yes		No	
Co-Morbidity	Yes		No	
If Yes, please state the name of the disease.				

Participants: ______________

Principle Investigator: __________________

National University of Sciences & Technology (NUST) Sector H-12, Islamabad

Table S6: Ethical approval form.

- **Research Project Title:** Impact of KPCI, KPCE, KPCG Genetic Variant in Prostate, Cervical and Ovarian cancer.

Name of Principal Investigator	Dr. Maia Shabbir
Duration	24 Months
Name of School/Department	ASAB-NUST
IRB No.	10-2021-01/01

The project entitled above has been reviewed by the NUST Ethical Review Committee Meeting held on **October 25, 2021.** Keeping in view the following mentioned areas.

- Qualification and Expertise of the Principle Investigator.
- Proposed Goals of the Study
- Selection Criteria of the Subjects
- Informed Consent in local language if required
- Potential Problems
- Research Design and Methods
- Potential Benefits of the study
- Risks of the Study
- Assessment & Management of Risk
- Confidentiality & Conflict of Interest.

The Committee approves above entitled project on scale and criteria given below to be implemented before/during project execution.

- Safety measures of carcinogenic chemicals.
- Designated space/work place/rooms for the experimental work.
- Protection of other research students/lab staff/animals from chemical hazardous.

The Ethical Review Committee reserves the rights to re-review the project during the project execution to address the suggested guidelines.

Prof. Dr. Peter John
Deptt of Healthcare Biotechnology
Atta-ur-Rahman School of Applied
Biosciences (ASAB), NUST Islamabad

Prof. Dr. Peter John

President Ethical Review Committee
